# Eco-epidemiological screening of multi-host wild rodent communities in the UK reveals pathogen strains of zoonotic interest

**DOI:** 10.1016/j.ijppaw.2022.02.010

**Published:** 2022-03-12

**Authors:** Flavia Occhibove, Niall J. McKeown, Claire Risley, Joseph E. Ironside

**Affiliations:** a IBERS, Aberystwyth University, Aberystwyth, SY23 3DA, UK; b UK Centre for Ecology & Hydrology, Wallingford, Oxfordshire, OX10 8BB, UK

**Keywords:** *Babesia microti*, *Bartonella* sp., Fleas, Ixodid ticks, Wild rodents, Zoonoses

## Abstract

Wild rodent communities represent ideal systems to study pathogens and parasites shared among sympatric species. Such studies are useful in the investigation of eco-epidemiological dynamics, improving disease management strategies and reducing zoonotic risk. The aim of this study was to investigate pathogen and parasites shared among rodent species (multi-host community) in West Wales in an area where human/wildlife disease risk was not previously assessed. West Wales is predominantly rural, with human settlements located alongside to grazing areas and semi-natural landscapes, creating a critical human-livestock-wildlife interface. Ground-dwelling wild rodent communities in Wales were live-trapped and biological samples – faeces and ectoparasites – collected and screened for a suite of pathogens and parasites that differ in types of transmission and ecology. Faecal samples were examined to detect Herpesvirus, *Escherichia coli*, and *Mycobacterium microti*. Ticks and fleas were collected, identified to species based on morphology and genetic barcodes, and then screened for *Anaplasma phagocytophilum*, *Babesia microti*, *Borrelia burgdorferi* sensu lato, and *Bartonella* sp. All the pathogens and parasites screened pose a characteristic epidemiological challenge, such as variable level of generalism, unknown zoonotic potential, and lack of data. The results showed that the bank vole *Myodes glareolus* had the highest prevalence of all pathogens and parasites. Higher flea species diversity was detected than in previous studies, and at least two *Bartonella* species were found circulating, one of which has not previously been detected in the UK. These key findings offer new insights into the distribution of selected pathogen and parasites and subsequent zoonotic risk, and provide new baselines and perspectives for further eco-epidemiological research.

## Highlights

• *Ixodes trianguliceps* dominated tick species found on sampled rodent populations.
• A zoonotic *Babesia microti* strain was isolated in ticks parasitising UK rodents.
• High flea diversity varied seasonally, harbouring at least two *Bartonella* species.
• Candidatus *Bartonella rudovakii* was isolated for the first time in the UK.

## Graphical abstract

**Figure:**
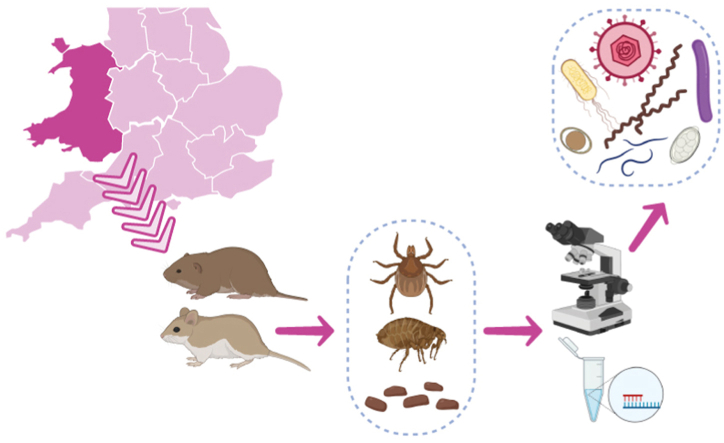

## 1 Introduction

Rodent communities represent ideal natural study systems to investigate the transmission of pathogens and parasites among sympatric host species (Begon et al., 1999; Telfer et al., 2007a; Paziewska et al., 2012). Usually, several rodent species with slightly different ecological niches share the same habitat and have overlapping home ranges, allowing interspecific transmission of parasites and pathogens. This allows multiple species to be surveyed relatively easily, using the same methodology (Wolton and Flowerdew, 1985). In the United Kingdom, the ground dwelling rodent community is dominated by wood mice (*Apodemus sylvaticus*), bank voles (*Myodes glareolus*), and field voles (*Microtus agrestis*) (Crawley, 1970; Greenwood, 1978). These species are considered the main reservoir hosts for a variety of pathogens, including Gammaherpesvirus (Knowles et al., 2012), Cowpox virus (Crouch et al., 1995) and *Bartonella* spp. (Birtles et al., 1994), and ecto-parasites, such as ticks and fleas (Whitaker, 2007). The various pathogens harboured by these rodent species vary in terms of transmission mode (i.e. direct (e.g. herpesvirus, cowpox virus) or vector-borne (e.g. *Bartonella* sp. through fleas, *Babesia microti* through ticks)), and have different degrees of generalism and affinities for host categories (e.g. sex, age, weight) (Walker et al., 2017). Furthermore, it appears that the role of each host species in transmission dynamics of generalist infectious agents is different (e.g. Begon et al., 1999), and not fully understood. As host species differ in abundance, exposure and susceptibility, it is likely that each species does not contribute equally to parasite transmission (Altizer et al., 2003). For example, the most frequently recorded Herpesvirus infecting wild rodent populations is Murid Herpesvirus 4 (Gammaherpesvirus) (Blasdell et al., 2003), which in the UK seems to present consistently higher prevalence in wood mice than bank voles (Telfer et al., 2007b).

Several rodent-borne pathogens also pose a zoonotic risk (Cleaveland et al., 2001), with the tick-borne *Borrelia burgdorferi* sensu lato, causative agent of Lyme disease in humans, being one of the most relevant and widespread zoonoses (Medlock and Leach, 2015). Rodent species harbour a remarkable proportion of zoonotic parasites, being one of the taxa with highest zoonotic potential (Olival et al., 2017), such that rodent-borne diseases represent a significant public health concern in many areas worldwide (Meerburg et al., 2009), including rural areas of Britain and Northern Europe. Rodents have a high probability of harbouring undiscovered zoonotic pathogens due to their life traits (e.g. early sexual maturity, high reproductive rate, large litters, rapid postnatal growth rate, small body size) (Han et al., 2015). Thus, understanding rodent-associated pathogens and parasites is crucial for disease-control policy, now and in the future because rodents are particularly resilient to environmental modifications and their numbers are predicted to rise as a consequence of defaunation of larger mammals (Young et al., 2014).

Rodent associated pathogens and parasites can also vary intraspecifically in their zoonotic potential. For example, *Escherichia coli*, is one of the most abundant bacteria associated with human and animal stool, but some strains are extremely pathogenic (e.g. shiga toxin-producing strains - STEC), and livestock and wildlife may act as reservoirs (Hughes et al., 2009). Rodent faecal samples, in Madagascar, were found to be almost three times more likely to carry *E. coli* than livestock, including strains found in human faeces while, in Europe, wildlife is known to harbour a much wider range of strains compared to humans (Bublitz et al., 2014). Another bacterium, *Mycobacterium microti*, the causative agents of vole tuberculosis (Brosch et al., 2002; van Soolingen et al., 1998), causes chronic, endemic infection in different species of wild British rodents, altering their population dynamics (Burthe et al., 2008; Cavanagh et al., 2002; Kipar et al., 2013; Turner et al., 2014). *Mycobacterium microti* has also been involved in infections in human subjects (Horstkotte et al., 2001; Niemann et al., 2000) and domestic animals (Emmanuel et al., 2007; Rüfenacht et al., 2011).

The most common ectoparasites in rodents are fleas and ticks, affecting host physiology, behaviour, survival, and population dynamics, depending on factors such as duration and intensity of infestation (Devevey and Christe, 2009; Hawlena et al., 2005; Hillegass et al., 2010), are recognised vectors of important zoonotic pathogens such as *B. burgdorferi* (Lyme disease), *Anaplasma phagocytophilum* (human granulocytic anaplasmosis), *Babesia* spp. (babesiosis), Flavivirus (tick-borne encephalitis), *Yersinia pestis* (plague), *Rickettsia* spp. (typhus), *Bartonella* spp. (bartonellosis) (Bitam et al., 2010; Dantas-Torres et al., 2012). Ecto-parasite infestation, or burden, varies considerably among individuals, and usually there is a high level of aggregation, determined by host individual characteristics (Brunner and Ostfeld, 2008) and environmental factors (e.g. Calabrese et al., 2011). Hard ticks (family Ixodidae) feed on the blood of a wide variety of vertebrates (Klompen et al., 1996), with small rodents being hosts of many different species (Paziewska et al., 2010). Understanding tick-host associations, and how multiple host species regulate tick dynamics, is very important to comprehend tick ecology, and predict patterns of tick distribution, especially in the context of tick and tick-borne disease management and control. Fleas (Insecta, Siphonaptera) feed on the blood on many higher vertebrates, preferring small burrowing mammals, and alternate between periods occurring on the host body and periods occurring in the host burrow (or nest) (Krasnov et al., 2002). As vectors, they harbour a large number of pathogens, of which the majority are still understudied, but can represent a serious threat in terms of emerging diseases (Bitam et al., 2010). In comparison with ticks, flea biology and ecology are poorly understood, flea-host relationship is still under investigation (Krasnov et al., 2015, 2016), and there have been relatively few studies of flea and flea-borne pathogen prevalence patterns, host-parasite dynamics and the role of fleas as pathogen vectors (Kowalski et al., 2015).

The tick-borne pathogens (Homer et al., 2000) *Anaplasma phagocytophilum* (bacterium of the order of Rickettsiales), *B. microti* (intraerythrocytic protozoan), and *Borrelia burgdorferi* sensu lato (spirochete bacterium) represent recognised zoonotic threats (Gray, 2006; Homer et al., 2000). Ixodid ticks can be simultaneously infected by these pathogens, but the dynamics of co-infection are not yet fully clear (Adelson et al., 2004; Hersh et al., 2014). *B. burgdorferi*, causative agent of Lyme disease, is one the most widespread and well-studied zoonotic tick-borne pathogens in temperate regions of North America, Europe, and Asia (Dantas-Torres et al., 2012; Kilpatrick et al., 2017a). Rodent fleas are vectors of numerous bartonellae, which have been implicated as the causative agents of human clinical manifestations, including endocarditis, myocarditis, fever and neurologic disorders, intraocular neuroretinitis, meningitis, splenomegaly, and lymphadenopathy (Gutiérrez et al., 2015; Tsai et al., 2011). In Britain, *Ctenophthalmus nobilis* collected from bank voles was confirmed to be an efficient vector of *Bartonella taylorii* and *Bartonella grahamii* (Bown et al., 2004), and other *Bartonella* species have been confirmed to circulate in woodland rodent communities, all of some zoonotic interest (Birtles et al., 2001; Telfer et al., 2007a,c).

The aim of the study was to provide additional knowledge on the epidemiology of rodent parasites and pathogen in the context of natural multi-host communities, as a step towards informing wildlife disease management and public health policy. More specifically, this study provides a description of the abundance and distribution of pathogen strains of zoonotic potential in an understudied system such as West Wales, an area where human/wildlife disease risk was not previously assessed. Although nationwide projects to monitor vectors and vector-borne pathogens exist (Abdullah et al., 2018; Big Tick Project and Big Flea Project by MSD Animal Health), these do not focus on wildlife and existing wildlife studies exist only for a restricted number of regions in the UK (e.g. Begon et al., 2009; Bown et al., 2003; Mallorie and Flowerdew, 1994; Marsh and Harris, 2000). Finally, we compared morphological identification and molecular barcoding of fleas and ticks in order to evaluate different molecular identification methodologies and contribute to the development of species identification resources. Our findings contribute to the understanding of rodent-borne pathogen and parasites distributions and subsequent zoonotic risk, and provide new perspectives for further eco-epidemiological research, particularly at the local scale.

## 2 Material and methods

### 2.1 Rodent live-trapping and ecto-parasite collection

West Wales is predominantly rural, with human settlements located alongside to grazing areas and semi-natural landscapes, creating a critical human-livestock-wildlife interface. Ground-dwelling wild rodent communities in Wales were screened for multiple pathogens and parasites, including ecto-parasites. The study received approval from the relevant Aberystwyth University ethical committee for research involving animals, the Animal Welfare and Ethical Review Body (AWERB). Rodents were live-trapped, and biological samples – faeces (fresh from the animal during handling or from the trap tunnel) and ecto-parasites – collected to allow the identification of pathogens and parasites differing in types of transmission and ecology. Trapping was performed in eight different sites in West Wales between June 2015 and June 2017. The sites were located in Ceredigion, mainland Pembrokeshire, and Skomer Island, and included a range of different habitats (Table 1). At each location, a square grid of 36 trapping stations (6 × 6) was set 15 m apart in woodlands and 7 m in grasslands, including one Longworth and one Sherman trap at each station (Anthony and Ribic, 2005). The traps were set up with appropriate bedding material and food; each trapping occasion consisted of three consecutive days and nights, following an initial day/night of pre-baiting, and the traps were checked twice a day (early morning and sunset). Trapping was performed twice for each year and site, once in the pre-breeding recruitment population phase (May–June) and a second time in the post-breeding peak population phase (September–October) to estimate individual densities in different seasons (excluding 2017, when the trapping only took place in the first season). Each individual, at the time of first capture, was identified at species level, sexed, assigned to an age class according to Telfer et al. (2002), weighed, individually marked by fur clipping, and finally released without any form of sedation. Biological samples – faeces and ecto-parasites – were collected from each individual only at first capture, and stored at −18 °C in sample tubes filled with RNAlater or at −80 °C without RNAlater to allow further molecular investigations. Traps were not a permanent feature of the environment and were removed between each trapping session. After each session all the traps were washed and disinfected with Virkon® or autoclaved to avoid cross-contamination between sites or seasons.

**Table 1:** List of habitat sampled during the study in two regions in Wales (UK). More details about the study sites and the vegetation communities in Occhibove (2018).

Region	Habitat
Ceredigion	Grassland/scrubland (coniferous forest clear-cut)
Ceredigion	Semi-deciduous natural woodland
Ceredigion	Coniferous woodland (grazed)
Pembrokeshire	Dune grassland
Pembrokeshire	Grassland/hedgerow
Pembrokeshire	Semi-deciduous natural woodland
Pembrokeshire	Semi-deciduous mixed forest (grazed)
Skomer Island	Bracken forest

Ecto-parasites, namely ticks and fleas, were collected from all small rodents sampled at the time at first capture. Ticks (Arachnida, Ixodida) were collected, after visual inspection, with fine point forceps mainly from animals’ cephalic area (Hussein, 1980; Randolph, 1975a). Fleas (Insecta, Siphonaptera) were collected according to McCauley et al. (2008) and Young et al. (2014). Each individual was held over an open, deep and transparent, plastic bag and then combed for 10 strokes with a flea comb; all the fleas recovered from both the bag and the comb, were collected.

Individual density of each species in each site, and for each trapping season was estimated by the POPAN algorithm (Schwarz and Arnason, 1996) within the software MARK (White and Burnham, 1999), assuming open population, constant survival, and constant capture probability. Goodness-of-fit of the best model selected by the software was tested with the RELEASE suite within the same software.

### 2.2 Diversity and prevalence of ecto-parasites

Frozen specimens were incubated with Dietrich's fixative solution overnight at 4 °C in 2 ml sample tubes and later identified under a compound microscope. Adult ticks were identified according to Hillyard (1996), while larval ticks were identified using Snow (1978). Life stage and sex of the adults was also recorded. Fleas were identified, when possible, at subspecies level according to Whitaker (2007). Ticks and fleas are not easy to identify morphologically, but barcoding techniques have not been fully validated yet, largely due to the lack of genomic sequences in publicly available databases (Pagel Van Zee et al., 2007), hence our application of molecular barcoding methods as an alternative identification method.

DNA extraction from ecto-parasite samples was performed through alkaline digestion (Bown et al., 2003). From each tick and flea genomic DNA sample ∼710 bp of the mitochondrial COI gene was amplified and sequenced using primers and the PCR protocol from Folmer et al. (1994). An ∼460 bp segment of the 16S gene was amplified and sequenced according to Black and Piesman (1994) and Bown et al. (2006) for ticks and Whiting (2001) for fleas. Initial identification of DNA sequences was performed using BLAST to search for similar sequences in the Genbank database, with sequence divergence of less than 2% indicating a likely species match (sequence identity >98%). In order to confirm the phylogenetic position of the ticks obtained from Welsh rodents, an alignment of concatenated COI and 16S sequences was generated using CLUSTAL, implemented in Mega 7 (Kumar et al., 2015) for the tick genus *Ixodes*, including sequences from Genbank that represented the major clades within this genus for which comparable sequences were available. With the addition of appropriate outgroup sequences from non-*Ixodes* ticks, this alignment was used to generate a phylogenetic tree using the Bayesian inference criterion implemented in MRBayes (Ronquist et al., 2012), using the general time reversible model with a proportion of invariant sites, identified as the model of best fit according to the Akaike information criterion (AIC). New DNA sequences were deposited in Genbank (Accession numbers are listed in Table 2).

**Table 2:** List of new sequences deposited in Genbank with relative accession numbers.

Genbank accession number	Organism
	Ticks
**MZ892385**	*Ixodes trianguliceps*
**MZ892599**	*Ixodes trianguliceps*
**MZ892600**	*Ixodes trianguliceps*
**MZ892601**	*Ixodes trianguliceps*
**MZ892602**	*Ixodes trianguliceps*
**MZ892610**	*Ixodes trianguliceps*
**MZ892611**	*Ixodes trianguliceps*
	Fleas
**MZ905355**	*Ctenophthalmus nobilis*
**MZ905356**	*Amalaraeus penicilliger*
**MZ905357**	*Amalaraeus penicilliger*
**MZ905458**	*Ctenophthalmus nobilis*
**MZ905459**	*Ctenophthalmus nobilis*
**MZ905460**	*Amalaraeus penicilliger*
**MZ905460**	*Hystrichopsylla talpae*
**MZ905461**	*Amalaraeus penicilliger*
**MZ905462**	*Doratopsylla dasycnema*
	Pathogens
**MZ892614**	*Anaplasma phagocytophilum*
**MZ901176**	*Bartonella grahamii*
**MZ901177**	*Bartonella rudakovii*-like
**MZ901374**	*Babesia microti*
**MZ934649**	*Escherichia coli*
**MZ934650**	Herpesvirus
**MZ934651**	Herpesvirus
**MZ934652**	Herpesvirus
**MZ934653**	Herpesvirus

Prevalence of ecto-parasites was analysed using generalised linear models, fitting a negative binomial distribution, according to Alexander (2012). It was investigated whether there was a significant difference among host species, host sex, sampling site, and sampling season. Data analyses were performed using the function glm.nb of the package MASS (Venables and Ripley, 2002) in R (R Core Team, 2020).

### 2.3 Pathogen screening

Total DNA was extracted from faecal samples using QIAamp DNA Stool Mini Kit (Qiagen, UK) according to the manufacturer's protocol. Faecal samples were screened by PCR for Herpesvirus, *E. coli*, and *Mycobacterium microti*. Ticks were screened for *Anaplasma phagocytophilum* (bacterium of the order of Rickettsiales), *Babesia microti* (intraerythrocytic protozoan), and *Borrelia burgdorferi* s.l. (spirochete bacterium). Fleas were screened for *Bartonella* sp. Details of these methodologies are presented in Table 3. Tick samples screened for pathogens were pooled according to site, season, and host to increase likelihood of pathogen detection. Species identification was performed using BLAST and phylogenetic tree inference as described previously. In order to confirm the phylogenetic positions of *Bartonella* sp. obtained from fleas on Welsh rodents and the *Babesia* sp. obtained from ticks on Welsh rodents, alignments of 18S sequences were obtained were generated including outgroup sequences from non-rodent *Bartonella* species (from moles and shrews) and *Babesia* species from outside the *B. microti* complex. Phylogenetic trees constructed using the Bayesian inference criterion implemented in MRBayes (Ronquist et al., 2012), using the Kimura 2 parameter model, the model of best fit according to the Akaike information criterion (AIC). New sequences were deposited in Genbank (Accession numbers are listed in Table 2).

**Table 3:** PCR methodology description for the amplification of pathogens DNA. Full description of the protocols and primers sequences available in the references provided unless specified differently.

Sample	Pathogen	PCR protocol	Primers	Reference	Positive control
Faecal	Herpesvirus	Nested PCR targeting the highly conserved DNA polymerase (DPOL) gene of Herpesviruses	ILK, DFA, TGV KG1, IYG	Vandevanter et al. 1996 Zheng et al., 2016	Clinical Virology Multiplex I: Immunodeficiency panel working reagent for Nucleic Acid Amplification Tests (NAT), from NIBSC
Faecal	*E. coli*	PCR targeting the malB promoter gene	ECO-1, ECO-2	Wang et al., 1996	Positive sample sequenced after pilot study
Faecal	*M. microti*	Nested PCR targeting the flanking regions of the RD1mic gene	RD1mic Fl Fw, RD1mic Fl Rv RD1mic Int Fw, RD1mic Int Rv	Brosch et al. (2002) Smith et al., 2009	Not available
Tick	*A. phagocytophilum*	Nested PCR targeting the 16S rDNA	ge3a, ge10r ge9f, ge2	Massung et al., 1998	Samples from Prof Richard Wall (University of Bristol)
Tick	*B. microti*	PCR specific for English strains (16S) PCR reaction targeting the 16S rDNA gene	KebabF, KebabR Bab1, Bab4	Bown et al. (2008) Schwartz et al. (1997)	Samples from Prof Richard Wall (University of Bristol)
Tick	*B. burgdorferi*	PCR targeting 23S rDNA	Bb23Sf, Bb23Sr	Courtney et al., 2004	Samples from Prof RichardWall (University of Bristol)
Flea	*Bartonella* sp.	Nested PCR that amplifies a fragment of the 16S–23S intergenic spacer region (ISR) PCR targeting the ssrA gene	big-F, big-R, bog-F ssrA-F, ssrA-R	Roux and Raoult 1995 Telfer et al. (2005) Diaz et al. 2012	Samples from Dr M. Kosoy (CDC, USA)

## 3 Results

### 3.1 Rodent community

The entire study comprised 4968 trap-nights with a total of 1195 captures (including recaptures). Captured individuals comprised *Apodemus sylvaticus* (wood mouse) (n = 230), *Myodes glareolus* (bank vole) (n = 258), and very few *Microtus agrestis* (field vole) (n = 9); on Skomer, the *M. glareolus* subspecies *M. g. skomerensis* (Skomer vole) was present (n = 183).

### 3.2 Diversity and prevalence of ecto-parasites

#### 3.2.1 Ticks

**Fig. 1:**
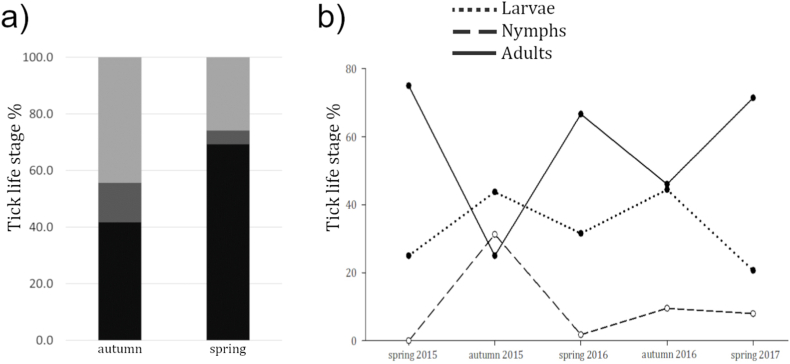
Percentage of tick life stages across seasons collected from all rodent species. a) Total percentage of ticks found in the two study seasons. Light grey: larvae; dark grey: nymphs; black: adults. b) Percentage of tick life stages in each sampling season.

In total, 225 ixodid ticks were collected from 120 rodents, 16.28% of total individuals sampled. The rodent hosts were of two species: *Apodemus sylvaticus* (wood mouse) and *Myodes glareolus* (bank vole). The median infestation (excluding zero values) was 1 for both species, as well as for the Skomer vole when data were analysed separately. Across all individuals sampled during the entire study, total infestation prevalence was 15.99%. Prevalence was higher in bank voles (18.14%) than wood mice (16.09%) (p < 0.01) (Table 4), and males exhibited a higher rate of infestation (21.74%) compared to females (13.17%) (p < 0.01) (Table 4). Bank voles resulted consistently more prevalent in each site, as well as males more heavily parasitised. Ticks were more prevalent in spring (27.18%) rather than in autumn (12.87%) (pooled data excluding Skomer Island because sampling there occurred only in one season) (p < 0.01). Adult ticks were more abundant in spring, while larvae and nymphs were more abundant in autumn (p < 0.01) (Fig. 1a and b).

**Table 4:** Average number of ticks per individual rodent. St.Dev: standard deviation; M: males; F: females. *p < 0.05.

Host species	Sex	Mean	St.Dev	Prevalence
**Bank vole**	M + F	0.38*	1.36	18.14*
	F	0.19	0.48	12.24
	M	0.23*	0.55	25.75*
**Wood mouse**	M + F	0.20	0.52	16.09
	F	0.28	1.28	16.45
	M	0.51	1.47	17.02

Regarding tick species, *Ixodes trianguliceps* was, by far, the most frequently represented in the sample set, being the most frequent species recovered on both host species (Table 5). This result was confirmed by DNA barcoding (see next section).

**Table 5:** Prevalence of tick species occurring on the sampled rodents according to morphological identification. In brackets sample size. Unknown species were specimen collected, but degraded to be identified by phenotypic features.

Species	Prevalence
*Ixodes acuminatus*	1.78 (4)
*Ixodes hexagonus*	1.33 (3)
*Ixodes ricinus*	4.44 (10)
*Ixodes trianguliceps*	84.00 (189)
*Ripicephalus sanguineus*	0.44 (1)
Unknown	8.00 (18)

Six mitochondrial COI DNA barcode sequences (434 bp) were obtained from ticks, all of which were identified as *Ixodes trianguliceps* based on morphological characters. The six sequences were identical and 99% similar to corresponding regions of the three published *I. trianguliceps* sequences available on Genbank (**MH784891** - **MH784893**), all of which had been isolated from *Myodes glareolus* from the Omsk region of Russia. Six 16S DNA barcode sequences (384 bp) were also obtained from ticks identified as *Ixodes trianguliceps* by morphology. These were also identical and 100% similar to corresponding regions of the three published *I. trianguliceps* sequences available on Genbank. A Bayesian phylogenetic tree of the concatenated COI and 16S sequences (Fig. S1) also supported the identification of these tick specimens as *I. trianguliceps*. In this case, the 16S marker alone would have been sufficient to confirm the identity of these ticks, due to the high genetic divergence of *I. trianguliceps* from other *Ixodes* species.

#### 3.2.2 Fleas

Over the entire study, 100 fleas were collected from 71 individuals, including representatives of all the rodent species trapped (i.e. bank vole, field vole, and wood mouse). The median infestation (excluding non-zero values) was 1 for all species (this was true for all sites and for both species, including the Skomer vole). Flea total prevalence was 8.70%, and there was no difference in prevalence between seasons, sites, or host sex. However, the wood mouse displayed significantly lower prevalence than the other species (p < 0.01) (Table 6).

**Table 6:** Average number of fleas per individual rodent and prevalence of infestation. St.Dev: standard deviation. *p < 0.05.

Host species	Mean	St.Dev	Prevalence
Bank vole	0.19*	0.63	12.24*
Field vole	0.55*	1.01	33.33*
Wood mouse	0.03	0.17	3.04

**Fig. 2:**
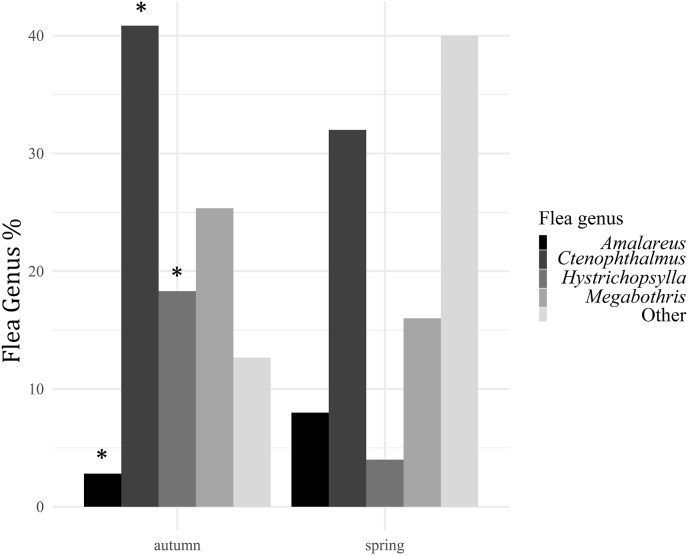
Flea diversity. Percentage of flea genera collected during the two sampling seasons. *p < 0.05.

In total, 12 species of fleas were morphologically identified, being two specimens recognised only at genus level (Table 7). *Ctenophtalmus* sp., *Megabothris* sp., and *Hystrichopsylla* sp. were more prevalent in autumn (p < 0.05) (fleas were grouped by genus for this analysis) (Fig. 2).

**Table 7:** Prevalence of flea taxa occurring on the sampled rodents according to morphological identification. In brackets sample size. Unknown species were specimen collected, but degraded to be identified by phenotypic features. *p < 0.05.

Taxonomic classification	Prevalence
*Amalareus penicilliger*	4.00 (4)
*Ctenophthalmus (Ctenophthalmus) nobilis*	26.00 (26)*
*Ctenophthalmus (Ctenophthalmus) nobilis vulgaris*	12.00 (12)*
*Ctenophthalmus* sp.	1.00 (1)
*Doratopsylla dasycnema*	1.00 (1)
*Hystrichopsylla talpae*	14.00 (14)*
*Leptopsylla (Leptopsylla) segnis*	1.00 (1)
*Megabothris (Gebiella) turbidus*	17.00 (17)*
*Megabothris (Megabothris) walkeri*	5.00 (5)
*Megabothris* sp.	1.00 (1)
*Nosopsyllus (Nosopsyllus) fasciatus*	1.00 (1)
*Nosopsyllus londiniensis*	2.00 (2)
*Peromyscopsylla spectabilis*	2.00 (2)
*Rhadinopsylla (Actenophthalmus) pentacantha*	2.00 (2)
*Typhloceras poppei*	1.00 (1)
Unknown	10.00 (10)

COI DNA barcode sequences (598 bp) were obtained from three fleas, all of which had been obtained from bank voles. Based on morphology, two of these fleas had been identified as *Amalaraeus penicilliger penicilliger* while the remaining specimen had been identified as *Ctenophthalmus nobilis nobilis*. The three sequences differed from one another by only a single base pair in one of the sequences and all were 99% similar to *Amalaraeus penicilliger penicilliger*, suggesting that one of the fleas had been incorrectly identified based on morphology. The 18S barcode sequences were obtained from nine fleas, identified by morphology as *Ctenophthalmus nobilis nobilis* and *Amalaraeus penicilliger penicilliger.* This region was sufficiently variable to confirm the identity of *C. n. nobilis*, due to its high divergence in this species. However, it was not sufficiently variable to allow confirmation of the identity of *A. a. penicilliger*. The 18S barcode sequences from the latter species precisely matched those of many flea species, including some from other genera.

### 3.3 Pathogen screening from faecal and ecto-parasite samples

In total, 358 faecal samples were analysed from the two sites with the highest individual rodent densities (a woodland on the Pembrokeshire mainland and Skomer Island). These were collected from autumn 2015 to autumn 2016; 299 samples were from bank voles (including 163 samples from the endemic Skomer vole subspecies *Myodes glareolus skomerensis*), and 59 samples were from wood mice. PCR screening of faecal samples detected Herpesvirus in 4/330 individuals, all of which were bank voles collected from Skomer (two adult females and two adult males). The sequences, of length ∼150 bp, showed overall similarity greater than >98% with human alphaherpesvirus 3 varicella-zoster (3 samples), and human alphaherpesvirus 2 herpes simplex (1 sample). The screen for *E. coli* produced a positive result from the faeces of 6/360 individuals, one adult male wood mouse and five adult bank voles (4 females and 1 male). However, the PCR screening was not entirely specific for *E. coli* and amplified other gut bacteria, such as Lachnospiraceae. The amplified region was not sufficiently variable to differentiate between strains of *E.* coli.

**Fig. 3:**
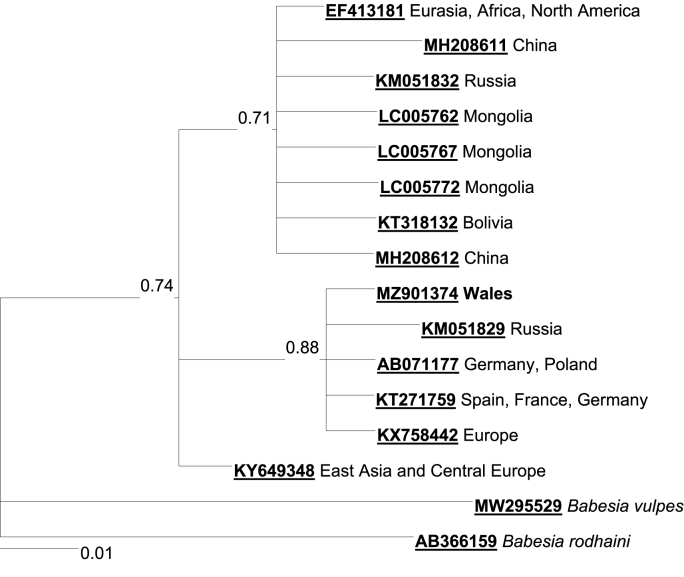
Bayesian phylogenetic tree of 18S ribosomal RNA sequences of *Babesia microti* isolates, indicating the position of the Munich strain-like isolate obtained from the tick *Ixodes trianguliceps* from a bank vole in Ceredigion, Wales. Sequences of the cogeneric species *B. vulpes* and *B. rodhaini* are used as outgroups.

*Anaplasma phagocytophilum* was detected by PCR in a single pooled sample of ticks collected from bank voles in spring at the woodland in Pembrokeshire. The sequence displayed high similarity (ID > 99%) with sequences available on Genbank from a wide variety of host species and locations but was not sufficiently variable to differentiate between strains. *Babesia microti* was detected by PCR in one sample of ticks collected from bank voles in spring (in a Ceredigion woodland). A phylogenetic tree places this sequence in a clade containing the ‘Munich’ strain of *Babesia microti*, together with sequences from small mammals from France, Spain, Germany, Poland and Russia (Fig. 3). The other two major clades consist predominantly of sequences from rodents from East Asia although both of these clades also contain small numbers of sequences from Europe and other parts of the World. While the ‘Munich’ strain has not been detected in the UK before using 18S primers, its presence in the UK was indicated previously by CCT*η* sequences obtained from two *M. glareolus* captured in the UK (Nakajima et al., 2009). This sample only showed amplification in the PCR performed with general *B. microti* primers (Schwartz et al., 1997), while specific primers for English strains (Bown et al., 2008) did not yield any PCR products (although positive control was amplified in both cases). No *B. burgdorferi* positive samples were found in this study.

*Bartonella* spp. were detected in fourteen fleas, twelve from bank voles, one from a field vole and one from a wood mouse. The infected flea species were represented by three *Amalaraeus penicilliger penicilliger*, five *Ctenophthalmus nobilis*, three *Hystrichopsylla talpae*, and three *Megabothris turbidus*. According to the results of the ssrA fragment, at least three strains of *Bartonella* were represented in the samples. These included one strain with a 100% sequence similarity to *B. grahamii* and another with 100% sequence similarity to Candidatus *Bartonella rudakovii* (Fig. S2), a species originally described from *M. glareolus* in Russia (Genbank **EF682088**) and also recorded from *M. oeconomus* in Lithuania (Mardosaitė-Busaitienė et al., 2019).

## 4 Discussion

This research undertook a targeted survey of the prevalence of different parasites and pathogens among rodent communities in different areas to provide insight the ecto-parasites and pathogens circulating among the understudied wild rodent communities in West Wales, with a particular focus on pathogens of potential zoonotic interest.

### 4.1 Rodent community

The species captured reflected the expected community assemblages in the habitats chosen. Wood mice (*Apodemus sylvaticus*) were found in all sampled sites, confirming the extreme generalism of this species (Millán de la Peña et al., 2003). However, slight demographic differences were found among species and sites, such as asynchronicity in abundance fluctuations, likely representing their asynchronicity in breeding peaks (Mallorie and Flowerdew, 1994; Huitu et al., 2004), which provides valuable information for improving epidemiological data collection on targeted species.

### 4.2 Ecto-parasites screening

Numbers of ecto-parasites recovered from rodents were in general agreement with other studies in which collection was made on living, non-anaesthetised individuals (e.g. Paziewska et al., 2010; Randolph, 1975a). Although collection of ecto-parasites from live individuals may underestimate the actual parasite burden compared to anesthetised or euthanised animals, it has been shown to permit an accurate characterisation of total ecto-parasite loads (Mooring and McKenzie, 1995); further, the collection method used for fleas has been proven to be a reliable indicator of flea population size (Krasnov et al., 2004).

The proportion of the population parasitised by ticks and fleas was small, supporting the “20/80 Rule” (Perkins et al., 2003; Woolhouse et al., 1997). Only 2.98% (22 individuals) were found infested with both ecto-parasites. In general, bank voles were more parasitised by ticks and fleas than wood mice in terms of prevalence, in agreement with Hussein (1980). In this study, *I. trianguliceps* accounted for 87% of the ticks collected, with only three cases of two species of ticks co-occurring on the same individual. The higher prevalence in bank voles may be due to the dominance of *I. trianguliceps*, which may prefer this species; instead, in areas where *I. ricinus* has been found to be the dominant tick species, higher prevalence in other small mammal species has been reported (e.g. Gray et al., 1999, Ireland; Kurtenbach et al., 1995; Germany). Fleas are also known to display species-specific host preferences (e.g. Young et al., 2014). In our study, bank voles were preferred to wood mice, although sharing the same flea species assemblage, as observed in other areas (e.g. Ireland: Telfer et al., 2005). Male-biased parasitism was found among ticks and fleas, as widely reported elsewhere (Krasnov et al., 2012), although overall host preference is also determined by other factors (Boyard et al., 2008; Brunner and Ostfeld, 2008; Randolph, 2004).

Ticks were more prevalent and abundant in spring, as expected according to their life cycle in the UK (Dobson et al., 2011; Randolph, 1975b, 2004; Randolph et al., 2002). A higher *I. trianguliceps* burden in spring was also found among small mammals in Norway, while *I. ricinus* displayed the opposite trend, being more abundant in autumn (Mysterud et al., 2015). This suggests niche segregation between the two species, a hypothesis that could not be investigated further in this study, but may be important to assess in the context of disease transmission in sites where different species of ticks can be found in sympatry. Fleas also seemed to displayed seasonality, as found in other temperate and tropical areas (e.g. McCauley et al., 2008, Kenya; Harris et al., 2009; Poland). Flea species are characterised by different reproductive strategies, being univoltine or bivoltine (i.e. single or double reproductive annual cycle respectively) (Harris et al., 2009). Our analyses, at genus level, revealed that *Hystrichopsylla*, *Ctenophthalmus*, and *Megabothris* were more prevalent in autumn (this result was consistent also when disaggregating the data by location). In Ireland, Telfer et al. (2007a) found that the flea community was more diverse in autumn, and dominated by *Peromyscopsylla spectabilis*, *H. talpae*, and *C. nobilis*. In this study, the spring flea community was more diverse and dominated by rare species. Nevertheless, *H. talpae*, *Ctenophthalmus* sp., and *Megabothris* sp. were significantly more prevalent in autumn, suggesting that these taxa might have a similar reproductive seasonality across Britain and Ireland. While further speculation on British reproductive strategies is not possible, this observation may be used to rethink the design of studies on flea diversity, host-flea assemblages, and flea-borne disease prevalence, which, in temperate areas, are usually suspended during winter, or are not continuous during the year. However, in light of our results, this design would likely miss considerable information; biotic and abiotic factors driving flea diversity and prevalence deserve further investigation to fully understand associated pathogen dynamics. In addition, in our study, in the context of flea diversity, a higher number of species were recovered compared to other field studies carried out in Britain and Ireland on native and invasive rodent species (e.g. Withenshaw et al., 2016; Telfer et al., 2007a).

Our results support previous findings that 16S and 18S genes give less phylogenetic resolution than COI in fleas and ticks (Hebert et al., 2003), but can be used as complementary to COI, when this latter fails (Lv et al., 2014). COI, which is the most frequently used fragment of animal DNA for barcoding, might still represent the best choice, but more research is needed to improve this approach, especially for obscure taxa, such as fleas (Lawrence et al., 2015). Currently, the combination of morphological and molecular approaches is still crucial.

### 4.3 Low prevalence of directly transmitted pathogens

The very low Herpesvirus prevalence found in this study might be explained by the type of sample tested, which might be considered not ideal. Despite individuals testing PCR positive for their entire lifetime when spleens and lungs are tested (Blasdell et al., 2003), these viruses can be latent for long periods, with the consequence that no viral particles are shed in the faeces (Nash et al., 2001). Likely for this reason, no wood mice were found infected in the populations sampled, although elsewhere the wood mouse was found to be the major reservoir host for Murine Herpesviruses (Telfer et al., 2007b). Rodents do not seem to represent reservoir hosts for pathogenic strains of *E. coli* (e.g. Healing et al., 1980; Kilonzo et al., 2013), which was in agreement with our findings (see also Swiecicka et al., 2003; Kozak et al., 2009). The presence of the bacterium may be related to gut microbiota variations due to diet seasonality (Ecke et al., 2018; Gebczynska, 1983; Hansson, 1979, 1985). No individuals were found infected by *M. microti*, likely due to the absence of lesions to the gastro-intestinal tract (Burthe et al., 2008; Cavanagh et al., 2002; Kipar et al., 2013). Wells (1946) discovered a high rate of *Mycobacterium* shedding in faeces and urine, associated with frequent lesions to gastro-intestinal and urinary tract. Ultimately, further investigations, possibly the analysis of liver and spleen, are needed to conclude that *M. microti* is absent from the populations sampled.

### 4.4 Prevalence of tick-borne pathogens

*A. phagocytophilum* was detected only at one site in ticks recovered on bank voles. Bown et al. (2003) also reported this species more likely to be infected compared to wood mice, probably due to a higher tick burden, as also noted in this study. The short infectious period may explain the low recovery of this pathogen (Bown et al., 2003), but it has also been proposed that the role of rodents as the main reservoirs of *A. phagocytophilum* should be reassessed (Baráková et al., 2018; Blaňarová et al., 2014; Bown et al., 2009; Burri et al., 2011). Only one tick sample was positive for the protozoan *B. microti*, although this has been often recorded in Britain in *I. trianguliceps* (Bown et al., 2008; Hussein, 1980). Low prevalence or absence has been reported in other studies (e.g. Healing, 1981; Welc-Falęciak et al., 2008), while high prevalence has been found in other species of rodents that might be the responsible for maintaining the enzootic cycle (Welc-Falęciak et al., 2008). Notably, and in line with Healing (1981), we did not recover *B. microti* from Skomer Island. The strain recovered in this study displayed high similarity to a European strain (Munich) involved in the first human case of *B. microti*-caused babesiosis (Arsuaga et al., 2016), suggesting that, in the UK, circulating *B. microti* strains differ in host and vector preferences, and in potential zoonotic risk (Gray, 2006). *Borrelia burgdorferi* s.l. was not found in the ticks collected, likely due to true absence or to *I. trianguliceps*, which accounted for more than 87% of the sampled ticks, not being a major vector of this spirochete (Kilpatrick et al., 2017; Stanek et al., 2012). Moreover, low infection prevalence in European rodents is considered part of a growing evidence that these may not be the main reservoir for Lyme disease in Europe (Chvostáč et al., 2018; Gray et al., 1999; Kurtenbach et al., 1998).

In summary, low prevalence of tick-borne pathogens recovered may be explained by true absence/low prevalence, low competence of rodent and vector populations sampled, negative impact on the pathogens tested of other undetected infections, or a combination of all these factors. Further investigations, such as longitudinal epidemiological studies on rodents instead of vectors are needed to improve the assessment of the potential zoonotic risk in the sampled areas.

### 4.5 Bartonella detection in the flea community

*Bartonella* spp. were detected in *A. penicilliger*, *M. turbidus*, *H. talpae*, and *C. nobilis*, the most common flea species among the ones collected. The overall prevalence of *Bartonella* spp. was in the range of values found in other field studies (e.g. Abbot et al., 2007; Abreu-Yanes et al., 2018; Stevenson et al., 2003; Withenshaw et al., 2016). In Ireland, Telfer et al. (2005) observed *Bartonella* sp. infection in a similar flea community to the one sampled here, and reported similar flea species prevalence. Flea prevalence seems not directly related to host infection (Withenshaw et al., 2016), but rodent host species displays seasonal prevalence variations connected with host demography and patterns of acquired immunity to different *Bartonella* species (Kosoy et al., 2004a; Telfer et al., 2007a). While this could not be tested in this study, no seasonality was recorded in flea infestation.

DNA sequencing indicated that at least two species, with high similarity to *B. grahamii*, and Candidatus *B. rudovakii*, were circulating at the sampling sites. *Bartonella grahamii* has been widely recorded in rodent fleas in the UK (Birtles et al., 2000; Telfer et al., 2007a, 2007c; Withenshaw et al., 2016) and other countries (e.g. Špitalská et al., 2017). *Bartonella rudovakii* was originally described from *M. glareolus* in Russia (Genbank **EF682088**), and has also been recorded from *M. oeconomus* in Lithuania (Mardosaitė-Busaitienė et al., 2019). To the best of our knowledge, this is the first time that *B. rudovakii* has been discovered in the UK.

## 5 Conclusion

In conclusion, our results indicate higher prevalence of pathogens and parasites in bank voles than in other rodent species, and higher levels of parasitism in male hosts than in females. The results on ecto-parasites are particularly interesting in the context of pathogen transmission because they shed some light on host preferences, vector assemblages, and vector seasonality. However, it is essential to investigate long-term local host-vector interaction dynamics to draw definitive conclusions on patterns of prevalence and intensity of infestation and identify individuals more likely to be involved in vector-borne disease transmission, assess disease risk, and developed targeted disease management strategies. In particular, flea biology and ecology are not fully understood yet and, in particular, the flea-host relationship is still under investigation (Krasnov et al., 2015, 2016), thus the data presented here are a valuable contribution to the uncovering of flea-host dynamics and flea role as pathogen vectors. Molecular screening and sequencing revealed at least two *Bartonella* species circulating in rodent fleas, of which one (Candidatus *B. rudakovii*) had not previously been detected in the UK, and also revealed that a *B. microti* strain, similar to the ‘Munich’ involved in the first case of human babesiosis, is circulating in UK rodent populations. These findings provide new perspectives for further eco-epidemiological research and have the potential to assist development of targeted strategies for disease risk management. Identification of general patterns in pathogen and parasite distribution and dynamics remains challenging; infectious disease distribution is uneven, with human zoonoses being particularly concentrated in some geographical areas, but the drivers of this phenomenon are not clear yet (Morand and Krasnov, 2010). Thus, from a wildlife management and public health perspective, it is essential to put more effort into wildlife population parasite and pathogen screening in order to enhance local and global eco-epidemiological understanding.

## Declaration of competing interest

The authors declare that they have no known competing financial interests or personal relationships that could have appeared to influence the work reported in this paper.
