# Supplementary material for: Eco-epidemiological screening of multi-host wild rodent communities in the UK reveals pathogen strains of zoonotic interest
Source: Int J Parasitol Parasites Wildl. 2022 Mar 12;17:278–87. doi: 10.1016/j.ijppaw.2022.02.010 (PMC8927908; doi:10.1016/j.ijppaw.2022.02.010)
Supplement: Supplementary file 1 [file mmc1.docx]

**Wild rodents eco-epidemiological screening study in the UK reveals pathogen strains of zoonotic interest**

Occhibove, Flavia^a,b*^, McKeown, Niall J.^a^, Risley, Claire^a^, Ironside, Joseph E^a^.

^a^IBERS, Aberystwyth University, Aberystwyth, SY23 3DA, UK

^b^UK Centre for Ecology & Hydrology, Wallingford, Oxfordshire, OX10 8BB, UK

*Corresponding author: [flaocc@ceh.ac.uk](mailto:flaocc@ceh.ac.uk), UK Centre for Ecology & Hydrology, Wallingford, Oxfordshire, OX10 8BB, UK; ORCID ID: https://orcid.org/0000-0001-7134-8416

**S1. Phylogenetic trees**

**Figure S1. Bayesian phylogenetic tree, indicating the position of the *Ixodes trianguliceps* concatenated Cytochrome Oxidase I and 16S rRNA sequences obtained from ticks on bank voles in Ceredigion and Pembrokeshire, Wales. The non-ixodes ticks *Ornithodoros hermsi* and *Argas persicus* are included as outgroups.**

***Ixodes trianguliceps* (UK)**

*Ixodes trianguliceps* (Russia)

1.00

*Ixodes ricinus*

*Ixodes holocyclus*

0.95

*Ixodes ovatus*

1.00

*Ornithodoros hermsi*

*Argas persicus*

0.1

**Figure S2. Bayesian phylogenetic tree of 18S ribosomal RNA sequences of rodent-infecting *Bartonella* species, indicating the position of the Candidatus *Bartonella rudovakii*-like species obtained from the flea *Amalaraeus penicilliger* *penicilliger* from a bank vole in Pembrokeshire, Wales. *Bartonella* sequences from non-rodent hosts are included as outgroups.**

**MF039563** *Bartonella* sp. (Slovakia)

**MF039562** *Bartonella* sp. (Slovakia)

1.00

**MF039578** *Bartonella* sp*.* (Slovakia)

0.96

**KU886457** *Bartonella* sp. (*Ixodes ricinus*, Germany)

**DQ155386** *Bartonella* sp. (*Apodemus agrarius*, Slovenia)

0.96

**KX267703** *Bartonella* sp. (*Ctenophthalmus agyrtes*, Slovakia)

**MF039574** *Bartonella* sp. (Slovakia)

0.99

**MF039576** *Bartonella* sp. (Slovakia)

**KU886454** *Bartonella* sp. (*Apodemus* spp., Germany, Slovakia, Slovenia)

0.55

**HM596449** *Bartonella* sp*.* (*Apodemus sylvaticus*, Spain)

0.54

**FN645485** *Bartonella* sp. (*Tamiasciurus hudsonicus*)

**KU292577** *Bartonella rochalimae* (*Procyon lotor*, USA)

1.00

**KU589255** *Bartonella* sp*.* (*Amalaraeus penicilliger*, UK)

1.00

**MZ901177 *Bartonella* sp. (*Amalaraeus penicilliger*, UK)**

**EF682087** *Bartonella rudakovii* (*Myodes glareolus*, Russia)

1.00

0.97

**MN244638** *Bartonella* sp. (*Arvicola sp*., Belgium)

0.96

**MN056393** *Bartonella* sp. (*Microtus arvalis*, Czech Republic)

**MN056392** *Bartonella* sp. (*Microtus arvalis*, Czech Republic)

0.99

0.52

1.00

**HM596451** *Bartonella* sp. (*Talpa europaea*, Spain)

**HM596450** *Bartonella* sp. (*Sorex coronatus*, Spain)

0.1
